# Supplementary material for: Effects of Long-Term Low-Protein Diets Supplemented with Sodium Dichloroacetate and Glucose on Metabolic Biomarkers and Intestinal Microbiota of Finishing Pigs
Source: Animals (Basel). 2022 Sep 21;12(19):2522. doi: 10.3390/ani12192522 (PMC9558518; doi:10.3390/ani12192522)
Supplement: Supplementary file 1 [file animals-12-02522-s001.zip › animals-1907839-supplementary.pdf]

Table S1. The ingredients and nutrient levels of diets for pigs with body weight ranging from 25 to 45 kg (dry matter basis, %).

Items	Diets				
	CON	LP	LP + DCA	LP + GLUC	LP + DCA + GLUC
Ingredients, %					
Corn	62.00	73.85	73.85	71.75	71.75
Soybean meal	25.10	12.77	12.77	13.27	13.27
Wheat bran	7.80	7.39	7.39	7.39	7.39
Fat powder[1]	2.00	2.00	2.00	2.00	2.00
L-Lys HCl, 78%	0.18	0.55	0.55	0.55	0.55
DL-Met, 99%	0.05	0.12	0.12	0.12	0.12
L-Thr, 98.5%	0.01	0.24	0.24	0.24	0.24
L-Trp, 98%	0.00	0.08	0.08	0.08	0.08
$CaHPO_4 \cdot 2H_2O$	0.69	0.90	0.90	0.90	0.90
$CaCO_3$	0.87	0.80	0.80	0.80	0.80
NaCl	0.30	0.30	0.30	0.30	0.30
Sodium dichloroacetate	0.00	0.00	0.012	0.00	0.012
Glucose	0.00	0.00	0.00	1.80	1.80
Premix[2]	1.00	1.00	1.00	1.00	1.00
Total	100.00	100.00	100.00	100.00	100.00
Nutrient levels, %					
ME[3], MJ/kg	13.9	13.9	13.9	13.9	13.9
Crude protein[4]	17.9	14.0	14.0	14.0	14.0
Ca[4]	0.62	0.61	0.61	0.61	0.61
P[4]	0.50	0.50	0.50	0.50	0.51
Lys[5]	0.94	0.94	0.94	0.94	0.94
Met[5]	0.32	0.32	0.32	0.32	0.32
Thr[5]	0.60	0.60	0.60	0.59	0.59
Trp[5]	0.22	0.22	0.22	0.22	0.22
Arg[5]	0.77	0.56	0.56	0.55	0.55
His[5]	0.44	0.33	0.33	0.33	0.33
Ile[5]	0.55	0.41	0.41	0.40	0.40
Leu[5]	1.05	0.83	0.83	0.81	0.81
Val[5]	0.73	0.57	0.57	0.56	0.56
Phe[5]	0.78	0.61	0.61	0.59	0.59
Pro[5]	0.73	0.56	0.56	0.55	0.55
Cys[5]	0.23	0.19	0.19	0.19	0.19
Asx (Asp+Asn)[5]	1.76	1.34	1.34	1.32	1.32
Ser[5]	0.56	0.41	0.41	0.40	0.40
Glx (Glu+Gln)[5]	2.46	1.82	1.82	1.79	1.79
Gly[5]	0.62	0.49	0.49	0.48	0.48
Ala[5]	0.67	0.52	0.52	0.51	0.51
Tyr[5]	0.34	0.26	0.26	0.26	0.26

[1]Fat powder provided is HTL-306 product (BergaFat, Berg + Schmidt Asia Pte Ltd, Singapore) which is fractionated palm fat combined with 6% selected phospholipids and free of trans fatty acids, supplying practically 100% energy in the form of fat with 36.82 MJ/kg digestible energy.

[2]Premix provided the following per kg diet: Cu (as copper sulfate), 100 mg; Fe (as ferrous sulfate), 100 mg; Zn (as zinc oxide), 120 mg; Mn (as manganese sulfate), 20 mg; I (as calcium

iodate), 0.3 mg; and Se (as sodium selenite), 0.3 mg; vitamin A, 3, 800 IU; vitamin D3, 800 IU; vitamin E, 10 IU; vitamin K, 1 mg; choline, 200 mg; pantothenic, 5 mg; vitamin B2, 2 mg; folic acid, 0.8 mg; niacin, 10 mg; vitamin B1, 1 mg; vitamin B6, 1 mg; biotin, 0.08 mg; vitamin B12, 0.01 mg.

[3]ME, metabolizable energy, calculated values.

[4]Analyzed values according to AOAC (2000).

[5]Values for standardized ileal digestible concentrations of amino acids in diets were calculated using standardized ileal digestible coefficients for the various ingredients provided by NRC (2012).

Table S2. The ingredients and nutrient levels of diets for pigs with body weight ranging from 45 to 65 kg (dry matter basis, %).

Items	Diets				
	CON	LP	LP + DCA	LP + GLUC	LP + DCA + GLUC
Ingredients, %					
Corn	65.00	76.00	76.00	73.70	73.70
Soybean meal	22.08	10.57	10.57	11.07	11.07
Wheat bran	7.80	7.39	7.39	7.39	7.39
Fat powder[1]	2.00	2.00	2.00	2.00	2.00
L-Lys HCl, 78%	0.16	0.50	0.50	0.50	0.50
DL-Met, 99%	0.04	0.10	0.10	0.10	0.10
L-Thr, 98.5%	0.00	0.22	0.22	0.22	0.22
L-Trp, 98%	0.00	0.07	0.07	0.07	0.07
$CaHPO_4 \cdot 2H_2O$	0.72	1.00	1.00	1.00	1.00
$CaCO_3$	0.90	0.85	0.85	0.85	0.85
NaCl	0.30	0.30	0.30	0.30	0.30
Sodium dichloroacetate	0.00	0.00	0.012	0.00	0.012
Glucose	0.00	0.00	0.00	1.80	1.80
Premix[2]	1.00	1.00	1.00	1.00	1.00
Total	100.00	100.00	100.00	100.00	100.00
Nutrient levels, %					
ME[3], MJ/kg	13.9	13.9	13.9	13.9	13.9
Crude protein[4]	16.6	13.2	13.2	13.2	13.2
Ca[4]	0.64	0.61	0.61	0.61	0.61
P[4]	0.48	0.48	0.48	0.48	0.48
Lys[5]	0.86	0.85	0.85	0.83	0.83
Met[5]	0.30	0.30	0.30	0.30	0.30
Thr[5]	0.57	0.57	0.57	0.56	0.56
Trp[5]	0.19	0.19	0.19	0.19	0.19
Arg[5]	0.70	0.56	0.56	0.55	0.55
His[5]	0.40	0.31	0.31	0.30	0.30
Ile[5]	0.49	0.39	0.39	0.38	0.38
Leu[5]	0.92	0.72	0.72	0.71	0.71
Val[5]	0.64	0.55	0.55	0.54	0.54
Phe[5]	0.68	0.54	0.54	0.51	0.51
Pro[5]	0.63	0.50	0.50	0.50	0.50
Cys[5]	0.20	0.14	0.14	0.14	0.14
Asx (Asp+Asn)[5]	1.55	1.21	1.21	1.19	1.19

Ser[5]	0.49	0.39	0.39	0.38	0.38
Glx (Glu+Gln)[5]	2.13	1.78	1.78	1.75	1.75
Gly[5]	0.55	0.42	0.42	0.41	0.41
Ala[5]	0.61	0.47	0.47	0.46	0.46
Tyr[5]	0.30	0.24	0.24	0.24	0.24

[1]Fat powder provided is HTL-306 product (BergaFat, Berg + Schmidt Asia Pte Ltd, Singapore) which is fractionated palm fat combined with 6% selected phospholipids and free of trans fatty acids, supplying practically 100% energy in the form of fat with 36.82 MJ/kg digestible energy.

[2]Premix provided the following per kg diet: Cu (as copper sulfate), 100 mg; Fe (as ferrous sulfate), 100 mg; Zn (as zinc oxide), 120 mg; Mn (as manganese sulfate), 20 mg; I (as calcium iodate), 0.3 mg; and Se (as sodium selenite), 0.3 mg; vitamin A, 3, 800 IU; vitamin D3, 800 IU; vitamin E, 10 IU; vitamin K, 1 mg; choline, 200 mg; pantothenic, 5 mg; vitamin B2, 2 mg; folic acid, 0.8 mg; niacin, 10 mg; vitamin B1, 1 mg; vitamin B6, 1 mg; biotin, 0.08 mg; vitamin B12, 0.01 mg.

[3] ME, metabolizable energy, calculated values.

[4]Analyzed values according to AOAC (2000).

[5]Values for standardized ileal digestible concentrations of amino acids in diets were calculated using standardized ileal digestible coefficients for the various ingredients provided by NRC (2012).

Table S3. The ingredients and nutrient levels of diets for pigs with body weight ranging from 65 to 95 kg (dry matter basis, %).

Items	Diets				
	CON	LP	LP + DCA	LP + GLUC	LP + DCA + GLUC
Ingredients, %					
Corn	72.44	82.11	82.10	79.80	79.80
Soybean meal	17.30	6.40	6.40	6.90	6.90
Wheat bran	6.00	6.00	6.00	6.00	6.00
Fat powder[1]	1.28	1.60	1.60	1.60	1.60
L-Lys HCl, 78%	0.07	0.44	0.44	0.44	0.44
DL-Met, 99%	0.02	0.09	0.09	0.09	0.09
L-Thr, 98.5%	0.00	0.20	0.20	0.20	0.20
L-Trp, 98%	0.00	0.06	0.06	0.06	0.06
$CaHPO_4{\cdot}2H_2O$	0.64	0.90	0.90	0.90	0.90
$CaCO_3$	0.95	0.90	0.90	0.90	0.90
NaCl	0.30	0.30	0.30	0.30	0.30
Sodium dichloroacetate	0.00	0.00	0.012	0.00	0.012
Glucose	0.00	0.00	0.00	1.80	1.80
Premix[2]	1.00	1.00	1.00	1.00	1.00
Total	100.00	100.00	100.00	100.00	100.00
Nutrient levels, %					
ME[3], MJ/kg	14.2	14.2	14.2	14.2	14.2
Crude protein[4]	15.0	11.7	11.7	11.7	11.7
Ca[4]	0.64	0.61	0.61	0.61	0.61
P[4]	0.50	0.50	0.50	0.51	0.51
Lys[5]	0.79	0.79	0.79	0.79	0.57

Met[5]	0.27	0.27	0.27	0.26	0.26
Thr[5]	0.51	0.51	0.51	0.51	0.51
Trp[5]	0.16	0.16	0.16	0.16	0.16
Arg[5]	0.60	0.47	0.47	0.46	0.46
His[5]	0.34	0.26	0.26	0.26	0.26
Ile[5]	0.42	0.32	0.32	0.32	0.32
Leu[5]	0.79	0.61	0.61	0.60	0.60
Val[5]	0.54	0.44	0.44	0.43	0.43
Phe[5]	0.58	0.45	0.45	0.44	0.44
Pro[5]	0.53	0.41	0.41	0.40	0.40
Cys[5]	0.17	0.13	0.13	0.13	0.13
Asx (Asp+Asn)[5]	1.32	1.02	1.02	1.00	1.00
Ser[5]	0.41	0.32	0.32	0.32	0.32
Glx (Glu+Gln)[5]	1.81	1.42	1.42	1.40	1.40
Gly[5]	0.45	0.34	0.34	0.34	0.34
Ala[5]	0.52	0.39	0.39	0.38	0.38
Tyr[5]	0.25	0.19	0.19	0.19	0.19

[1]Fat powder provided is HTL-306 product (BergaFat, Berg + Schmidt Asia Pte Ltd, Singapore) which is fractionated palm fat combined with 6% selected phospholipids and free of trans fatty acids, supplying practically 100% energy in the form of fat with 36.82 MJ/kg digestible energy.

[2]Premix provided the following per kg diet: Cu (as copper sulfate), 100 mg; Fe (as ferrous sulfate), 100 mg; Zn (as zinc oxide), 120 mg; Mn (as manganese sulfate), 20 mg; I (as calcium iodate), 0.3 mg; and Se (as sodium selenite), 0.3 mg; vitamin A, 3, 800 IU; vitamin D3, 800 IU; vitamin E, 10 IU; vitamin K, 1 mg; choline, 200 mg; pantothenic, 5 mg; vitamin B2, 2 mg; folic acid, 0.8 mg; niacin, 10 mg; vitamin B1, 1 mg; vitamin B6, 1 mg; biotin, 0.08 mg; vitamin B12, 0.01 mg.

[3]ME, metabolizable energy, calculated values.

[4]Analyzed values according to AOAC (2000).

[5]Values for standardized ileal digestible concentrations of amino acid in diets were calculated using standardized ileal digestible coefficients for the various ingredients provided by NRC (2012).
